# Get Noticed to Get Ahead: The Impact of Personal Branding on Career Success

**DOI:** 10.3389/fpsyg.2019.02662

**Published:** 2019-12-09

**Authors:** Sergey Gorbatov, Svetlana N. Khapova, Evgenia I. Lysova

**Affiliations:** School of Business and Economics, Vrije Universiteit Amsterdam, Amsterdam, Netherlands

**Keywords:** personal branding, self-presentation, employability, career, theory of planned behavior

## Abstract

There is a growing amount of attention being brought to personal branding as an effective career behavior, but little is known about the factors that predict personal branding behaviors and their outcomes. In two studies (*N* = 477) across two distinctly different cultural contexts (Western and Asian) based on a newly developed and validated scale of personal branding, we have examined the antecedents and outcomes of personal branding. The findings confirm that personal branding leads to greater career satisfaction, fully mediated by perceived employability. Career achievement aspiration was the strongest predictor of engaging in personal branding, while career feedback negatively related to personal branding intention and career self-efficacy positively related to personal branding but not to personal branding intention. These findings highlight the importance of personal branding as a contemporary career technique in promoting one’s personal brand identity to achieve beneficial career outcomes.

## Introduction

The contemporary employment environment and increased amount of flexible work arrangements require individuals to become much more market oriented (Lair et al., 2005; Manai and Holmlund, 2015). One concept that captures such personal marketing orientation is *personal branding*, which refers to “a strategic process of creating, positioning, and maintaining a positive impression of oneself, based in a unique combination of individual characteristics, which signal a certain promise to the target audience through a differentiated narrative and imagery” (Gorbatov et al., 2018, p. 6). Research shows that personal branding helps individuals to attain positive career outcomes, among which are social capital (Gandini, 2016; Paivi and Back, 2017; Tarnovskaya, 2017), financial rewards (Close et al., 2011; Rangarajan et al., 2017), and career opportunities (Parmentier et al., 2013; Schlosser et al., 2017).

Increasingly, individuals today do their work through the Internet (e.g., gig-work), where “self-branding in the knowledge economy is a device for self-promotion for the pursuit of self-realization” (Gandini, 2016, p. 124). This global trend of digitalization for many career seekers means an opportunity to offer their skills and competencies globally and across boundaries of industries and organizations. This is done through personal branding, or in other words, through making one’s individual value proposition known to the target audience. As a concept, personal branding comes from the marketing literature (Lair et al., 2005; Shepherd, 2005). Although it is still considered to be a new concept, there are already more than 100 papers published on the topic of personal branding in the organizational behavior literature, as evidenced by a recent literature review by Gorbatov et al. (2018). Yet, due to the paucity of quantitative empirical studies on personal branding, none of this research gives a clear answer to the question of what the antecedents and outcomes of personal branding are in the career context. Addressing this research gap is urgent and relevant given the growing number of individuals who engage in personal branding behaviors on the Internet and, specifically, social media.

In this paper, we aim to fill this research gap by developing and testing a model of antecedents and outcomes of personal branding in the Western and Asian cultural contexts. The main focus of this paper is to test the theoretical relationships between personal branding and other career constructs. As no validated measure of personal branding existed, we had to develop one. In doing so, we followed the approach of Carmeli et al. (2015), who created a measure of respective engagement as a preliminary step to empirical testing of their hypotheses. Consequently, we first developed a personal branding scale, and, in Study 1, we then cross-validated this measure and explored the relationship between personal branding and its outcomes (i.e., perceived employability and career satisfaction). In Study 2, building on the theory of planned behavior (Ajzen, 1991), we examined the antecedents of personal branding.

Thus, epistemologically, we extend the extant career theory to incorporate personal branding as an increasingly important career tool in the contemporary digitalized work environment. Second, we explore the ontology of the relationships between personal branding and other related concepts, such as career aspiration, employability, and career satisfaction. Finally, we make a methodological contribution by developing and validating a personal branding scale, enabling future research in the field.

## Theoretical Background

### Personal Branding

The concept of personal branding originated in marketing research (Keller, 1993; Keller and Lehmann, 2006) and since then entered the field of organizational and vocational studies as a type of proactive work behavior (Crant, 2000, p. 436). The definition of personal branding establishes it as a proactive work behavior that employs marketing strategies and tactics to achieve career benefits in three distinct ways: strategic, differentiated, and technology based. First, while some other self-presentation behaviors from the same nomological field, such as impression management, may be both conscious and unconscious (Bolino et al., 2016), personal branding is strategic, which means that the activities are coordinated and point in a defined direction, targeting a specific audience. Second, effective personal branding achieves differentiation of the marketed self, conveying valued and unique individual characteristics against the competition or the frame of reference. It signals benefits or communicates a promise to deliver an outcome valued by others, while fitting into the expectations of a field (Parmentier et al., 2013). In the studies of human behavior, this is known as “optimal distinctiveness,” or the competing needs for assimilation and inclusion and the need for differentiation from the in-group (Brewer, 1991; Leonardelli et al., 2010). Finally, personal branding today heavily relies on technology as the primary vehicle to convey imagery (e.g., logo, photos, and work samples) and related storytelling to the target audience. Textual and visual performances make personal branding tangible and real (Pera et al., 2016; Pagis and Ailon, 2017), resulting in a stream of studies examining the use of technology for personal branding, such as LinkedIn profile photos (van der Land et al., 2016; Tifferet and Vilnai-Yavetz, 2018), Facebook profiles (Labrecque et al., 2011), Instagram photos (Geurin-Eagleman and Burch, 2016), YouTube channels (Chen, 2013), academic portals ResearchGate and Mendeley (Van Noorden, 2014), and Twitter activity (Brems et al., 2017; Hedman, 2017). Technology also allows career seekers to estimate the effectiveness of personal branding activities, which is essential for sense making and applying any corrective measures when necessary.

In sum, personal branding as an intentional individual career behavior emerged in response to the increasing emergence of new communication technologies in all parts of people’s lives and work as well as the changes in the labor market and the employer-employee relationship (Vallas and Christin, 2018). In these new forms of employment, personal branding is an important factor of career success (Shepherd, 2005; Parmentier et al., 2013; Gioia et al., 2014) as an adaptable career behavior aimed at packaging and presenting one’s professional identity to meet the needs of the target audience.

### Personal Branding and Career Outcomes

Traditionally, career outcomes have been conceptualized as career success including largely objective and, to a lesser extent, subjective facets. As such, career success is defined as “the accomplishment of desirable work-related outcomes at any point in a person’s work experiences over time” (Arthur et al., 2005, p. 179). This traditional conceptualization of career outcomes is relevant for employees who work in a single company during their whole employment (Wang and Wanberg, 2017). Today, however, individuals move from firm to firm and from job to a job frequently, and they also find themselves in novel employment relationships, such as freelancing (van den Born and van Witteloostuijn, 2013; Kuhn, 2016), temporary and contract working conditions (Davis-Blake and Uzzi, 1993), and *recareering* or mid- and late-career changes (Wöhrmann et al., 2014; Rice, 2015; Robertson, 2017). Career outcome criteria other than objective career success are therefore more important to contemporary workers.

Career satisfaction is an important subjective career outcome, and it is shown to be the result of processes requiring agency in managing one’s career, such as career self-management (King, 2004), impression management (Cheng et al., 2014), and career adaptability (Rudolph et al., 2017). For example, studying 195 employee-supervisor dyads from various industries in Taiwan, Cheng et al. (2014) showed that individuals who employed self-promotion behaviors showed greater career satisfaction compared to those who did not employ such behaviors. Since personal branding and self-promotion are self-presentation behaviors, we hypothesized that personal branding would also be positively related to career satisfaction.

*Hypothesis 1a*: Personal branding is positively related to career satisfaction.

Perceived employability, defined as “one’s ability to identify and realize career opportunities” (Fugate et al., 2004, p. 23), is also considered to be one of the leading career outcomes of contemporary employees. In line with the employability research, and with its focus on the individual positive assessment of his/her marketability on the external and internal job markets, we propose that efforts made in promoting oneself through personal branding will lead to higher perceived employability. One of the central processes in personal branding is constructing the desired professional image of self, and there is evidence that clarity of professional self positively impacts employability (Lysova et al., 2018).

*Hypothesis 1b*: Personal branding is positively related to perceived employability.

Furthermore, perceived employability is expected to mediate the relationship between personal branding and higher career satisfaction. This is supported by recent findings that employability is positively related to career satisfaction. For example, studies have shown that career satisfaction is an outcome of both career adaptability (Rudolph et al., 2017) and a stronger sense of professional identity (McKevitt et al., 2017). The mediating effect of employability of the relationship between emotional self-efficacy and career satisfaction was examined, for example, by Dacre Pool and Qualter (2013), who found that employability mediated the relationship between emotional self-efficacy and career satisfaction. Besides, personal branding has a signaling function. By communicating one’s professional value, individuals can reduce the information asymmetry problem in the labor market to their advantage (Zinko and Rubin, 2015) to achieve the desired differentiation, as signaling is positively related with career success (Ramaswami et al., 2010). Finally, people who engage in personal branding have high social capital (Bourdieu, 1993); they engage in such activities as communicating their own value proposition or informing others of personal achievements. Social capital was found to be positively related to career success (Parmentier et al., 2013; Delisle and Parmentier, 2016; Caro Castaño, 2017). Seibert et al. (2001a,b) demonstrated how greater social capital in the form of access to information, access to resources, and career sponsorship leads to increased career satisfaction.

*Hypothesis 1c*: Perceived employability mediates the relationship between personal branding and career satisfaction.

To test the hypotheses, we developed a measurement of personal branding and conducted two studies. The purpose of Study 1 was to explore the relationship between personal branding, perceived employability, and career satisfaction, while cross-validating the new measurement instrument; we examined the antecedents of personal branding in Study 2.

## Personal Branding Measurement Development

### Method

We constructed the scales to measure personal branding, using the Likert method as described by Dawis (1987). Drawing from the construct definition, we collected a pool of 39 items (15 for strategic, 11 for differentiated, and 13 for technologically savvy) that were reviewed for clarity and content validity by an industrial and organizational psychologist and a marketing professor (the full list can be obtained from the corresponding author). All items were answered using a five-point Likert scale (1 = *strongly disagree*, 5 = *strongly agree*).

We recruited 1,001 participants on the Amazon Mechanical Turk platform, where they completed the survey for pay. Since Fokkema and Greiff (2017) advised against performing EFA and CFA on the same sample, we split the sample into two to perform the exploratory (EFA) and confirmatory (CFA) factor analyses separately according to employment status to establish that the scale works well for both workers and job seekers. We split the sample by the employment status of the respondents to examine whether the EFA and CFA results would be consistent across these different groups. The invariance analysis revealed that there were no statistically significant variances in the measurement model across the two groups.

#### Exploratory Factor Analysis

We conducted the EFA on the population of the sample who were not in full employment (*N* = 204): female = 54.9%; average age = 33; US (97 cases, 47.5%), India (60 cases, 29.4%), other countries not exceeding 5% of the total sample. The employment status of the respondents was as follows: employed, part time = 72.5%; not employed, looking for work = 14.7%; not employed, not looking for work = 7.4%; retired = 4.9%; disabled, not able to work = 0.5%. We used principal factor analysis with promax rotation (Osborne and Fitzpatrick, 2012) in SPSS to examine the potential factor structure of the scale. We iteratively removed the items with loadings <0.35 as well as items that cross-loaded >0.35 two or more factors.

#### Confirmatory Factor Analysis

CFA was performed on the employed population of the Mechanical Turk sample (*N* = 797): female = 44.5%; average age = 33; US = 56.2%, India = 35.8%, other countries not exceeding 2% of the total sample. In a conservative approach, we used seven indices to assess model fit (Noar, 2003; Schreiber et al., 2006): Chi-square/df ratio (*χ*^2^/df); relative fit indices—normed fit index (NFI), incremental fit index (IFI), Tucker Lewis index (TLI), comparative fit index (CFI), and parsimony-adjusted measures—root mean square error of approximation (RMSEA); and *p* of close fit (*p*_close_).

To demonstrate the equivalence of all items designed to measure personal branding across various samples, we performed invariance testing of the scale by analyzing the differences across genders in the unconstrained, constrained measurement weights, constrained structural covariances, and fully constrained models.

### Results

The EFA yielded a three-factor structure comprised of 18 items. The Cronbach’s alphas for the three factors were 0.80, 0.83, and 0.90—above the acceptable cut off point of 0.70 (Nunnally and Bernstein, 1994). Together, the three factors explained 58.7% of the variance, with correlations among them of 0.46, 0.53, and 0.61 (*p* < 0.001), supporting their distinctiveness.

The initial CFA on the employed part of the sample confirmed the three-factor model, and its fit indices were acceptable (*χ*^2^/df = 4.02; NFI = 0.92; IFI = 0.94; TLI = 0.93; CFI = 0.94; and RMSEA = 0.06, where *p*_close_ < 0.001). The standardized regression weights for all items were greater than 0.50. Table 1 summarizes the EFA and CFA outcomes.

**Table 1:** Personal branding scale items and their factor loadings.

Factor	Item	*λ*	*α*_1_	*α*_2_
Strategic	1. I purposefully engage in experiences that can enhance my professional image.	0.76	0.80	0.83
	2. I make an effort to expand my professional network.	0.72		
	3. I have established routines to communicate my professional image to my network.	0.68		
	4. I actively develop my professional image.	0.66		
	5. I proactively adjust my professional image to manage expectations of the target audience.	0.59		
	6. I am strategic in the type of information I communicate about myself.	0.54		
Differentiated	7. I proactively seek the endorsement of others to promote the quality of my work.	0.73	0.83	0.81
	8. I make an effort to have a distinct profile compared to others in my professional area.	0.68		
	9. I make my successes known to my professional network.	0.67		
	10. I make an effort to present myself differently from my peers.	0.64		
	11. I consistently communicate that I deliver valuable work.	0.58		
	12. I make sure that what I do is recognizable.	0.58		
Technologically savvy	13. I use data to estimate my impact on my professional network.	0.79	0.90	0.89
	14. I use online tools and metrics to evaluate how others see me professionally.	0.77		
	15. I systematically analyze the effectiveness of my personal branding activities.	0.76		
	16. I actively communicate about my professional activities on social media.	0.75		
	17. I ensure that my online educational and/or professional profiles are complete (informative, engaging, have photos).	0.74		
	18. I post online samples or descriptions of my work projects.	0.66		

*λ, standardized regression weight (all p’s < 0.001); α_1_, Cronbach’s alpha for the factor in the EFA study; α_2_, Cronbach’s alpha for the factor in the CFA study*.

As there is some evidence that women may engage in personal branding differently than men (Rui and Stefanone, 2013; Thompson-Whiteside et al., 2018), it was important to establish invariance of the scale across genders. The scalar invariance testing returned values of *p* greater than 0.05 in all instances when the measurement weights, structural covariances, and measurement residuals were constrained (Table 2). This allowed us to reject the null hypothesis that there are statistically significant variances in the measurement model across genders.

**Table 2:** Invariance testing.

Model	df	*χ*^2^	*p*	CFI	NFI	IFI	TLI
1. Unconstrained model				0.95	0.92	0.95	0.94
Measurement weights	15	14.86	0.46				
Structural covariances	21	26.36	0.19				
Measurement residuals	44	46.80	0.36				
2. Constrained measurement weights				0.95	0.92	0.95	0.95
Structural covariances	6	11.51	0.07				
Measurement residuals	29	31.94	0.32				
3. Constrained structural covariances				0.95	0.91	0.95	0.95
Measurement residuals	23	20.43	0.62				

*N = 797. CFI, comparative fit index; NFI, normed fit index; IFI, incremental fit index; TLI, Tucker Lewis index*.

Throughout the subsequent studies, we continue to establish the predictive validity of the personal branding scale.

## Study 1

The aim of this study was to examine the relationship between the outcome measures of personal branding, while testing the newly developed personal branding scale to establish its external validity.

### Method

#### Participants

We collected 306 responses *via* an online survey that was distributed by two master’s students at a large public university in the Netherlands to people in their networks (e.g., classmates, friends, professional contacts, etc.) in accordance with the research ethics regulations of that university. Completing the survey was anonymous and the participants could withdraw at any moment. After the initial visual and boxplot analyses, 43 responses were removed because of acquiescing responding (i.e., providing same values for all items) or missing values in the key variables of interest, which resulted in an analyzable sample of 263 cases (female = 58.6%; Mean Age = 27 (SD = 9.5); the Netherlands = 71.9%, China = 23.2%; employed part-time = 45.6%, employed full-time = 30%, not employed, not looking for work = 14.8%, not employed, looking for work = 8.4%; and 5 years of work experience or less = 65%).

#### Measures

Answers to all variables were given on a five-point scale ranging from 1 (*strongly disagree*) to 5 (*strongly agree*).

*Personal branding* was measured by the 18-item scale developed in this paper. Cronbach’s alpha for the overall scale was 0.87. The alphas for the three factors of the scale (strategic, differentiated, and technologically savvy) were 0.77, 0.73, and 0.83, respectively. As we were interested in overall personal branding behavior rather than its subfactors, we chose to stay at the higher scale level.

*Perceived employability* was measured with the five-item scale developed by Berntson and Marklund (2007). An example item was “My experience is in demand on the labor market.” Cronbach’s alpha was 0.76.

*Career satisfaction* was measured with a four-item scale by Turban and Dougherty (1994). An example item was “Given my age, my career is on or ahead of schedule.” Cronbach’s alpha was 0.83.

We used gender and age as control variables, given earlier findings that men and women approach personal branding differently (Lobpries et al., 2018; Thompson-Whiteside et al., 2018) and an assumption that there will be variance across generations in the abilities to strategically differentiate self in the labor market and the technological savvy to do so effectively online (Reisenwitz and Iyer, 2009).

#### Analytical Strategy

The analyses were performed in two steps using the AMOS software (Arbuckle, 2017). In the first step, the measurement model was tested. We performed a series of CFAs to establish the discriminant validity of the constructs in the model. In the second step, we used structural equation modeling (SEM) to test the theoretical model, using the maximum likelihood method of estimation. To assess the fit of the models, we used various measures: *χ*^2^/df, CFI, TLI, RMSEA, and SRMR (Browne and Cudeck, 1993; Hu and Bentler, 1999; Kline, 2016). To estimate the indirect effects, accounting for multivariate non-normality of the data, we used bootstrapping technique with 5,000 bootstrapping samples and 95% confidence intervals (Preacher et al., 2010; Kline, 2016). Bootstrapping does not assume the sampling distribution as normal and performs iterative resampling analyses, resulting in more accurate confidence intervals of indirect effects as it derives the estimates of the parameters of the model strictly from the sample (Preacher and Hayes, 2008).

### Results

#### Measurement Model

The measurement model, including three latent variables (i.e., personal branding, perceived employability, and career satisfaction), showed an acceptable fit to the data: *χ*^2^ = 529.40, df = 315, *χ*^2^/df = 1.68, *p* < 0.001, CFI = 0.91, TLI = 0.90, RMSEA = 0.05 (*p*_close_ = 0.41), and SRMR = 0.06. This model’s fit was better than the fit of the model where all the variables loaded on one latent factor (*χ*^2^ = 773.32, df = 318, *χ*^2^/df = 2.43, *p* < 0.001, CFI = 0.81, TLI = 0.78, RMSEA = 0.07 (*p*_close_ < 0.001), SRMR = 0.11, ∆*χ*^2^ = 243.92, df = 3, *p* < 0.001). All the items had significant loadings on the intended factors (range *λ* = 0.41–0.86, *p*’s < 0.001).

#### Descriptive Statistics

Means, standard deviations, and correlations among all the study variables are shown in Table 3. Contrary to our expectations, neither age nor gender had significant correlations with any of the dependent variables, and we therefore continued with the analyses without these measures. Personal branding was moderately correlated with perceived employability (*r* = 0.48, *p* < 0.01) and weakly correlated with career satisfaction (*r* = 0.28, *p* < 0.01), indicating a more distal relationship with the latter. As expected, perceived employability was significantly correlated with career satisfaction (*r* = 0.48, *p* < 0.01).

**Table 3:** Study 1 variables’ means, standard deviations (SD), and correlations.

	Mean	SD	1	2	3	4	5
1. Gender	1.59	0.49	—				
2. Age	27.25	9.49	−0.16^*^	—			
3. Personal branding	3.22	0.53	−0.07	0.07	**0.87**		
4. Perceived employability	3.50	0.60	−0.05	0.06	0.48^**^	**0.76**	
5. Career satisfaction	3.52	0.74	0.01	0.04	0.28^**^	0.48^**^	**0.83**

*N = 263. Cronbach’s alphas are displayed in bold on the diagonal*.

* p < 0.05;

** *p < 0.01*.

#### Hypotheses Testing

The mediation model where personal branding influences career satisfaction *via* perceived employability showed an acceptable fit to the data: *χ*^2^ = 529.40, df = 315, *χ*^2^/df = 1.68, *p* < 0.001, CFI = 0.91, TLI = 0.90, RMSEA = 0.05 (*p*_close_ = 0.41), SRMR = 0.06. We tested two alternative models: a full mediation model and a model where perceived employability impacts career satisfaction *via* personal branding (i.e., personal branding is a mediator). The full mediation model was not significantly different from the baseline partial mediation one: *χ*^2^ = 530.70, df = 316, *χ*^2^/df = 1.68, *p* < 0.001, CFI = 0.91, TLI = 0.90, RMSEA = 0.05 (*p*_close_ = 0.41), SRMR = 0.06, ∆*χ*^2^ = 1.3, df = 1, *p* = 0.254. The model with personal branding as a mediator showed a poorer fit: *χ*^2^ = 575.82, df = 316, *χ*^2^/df = 1.82, *p* < 0.001, CFI = 0.89, TLI = 0.88, RMSEA = 0.06 (*p*_close_ = 0.08), SRMR = 0.07, ∆*χ*^2^ = 46.42, df = 1, *p* < 0.001. We therefore proceeded with the analyses on the baseline model.

**Figure 1:**
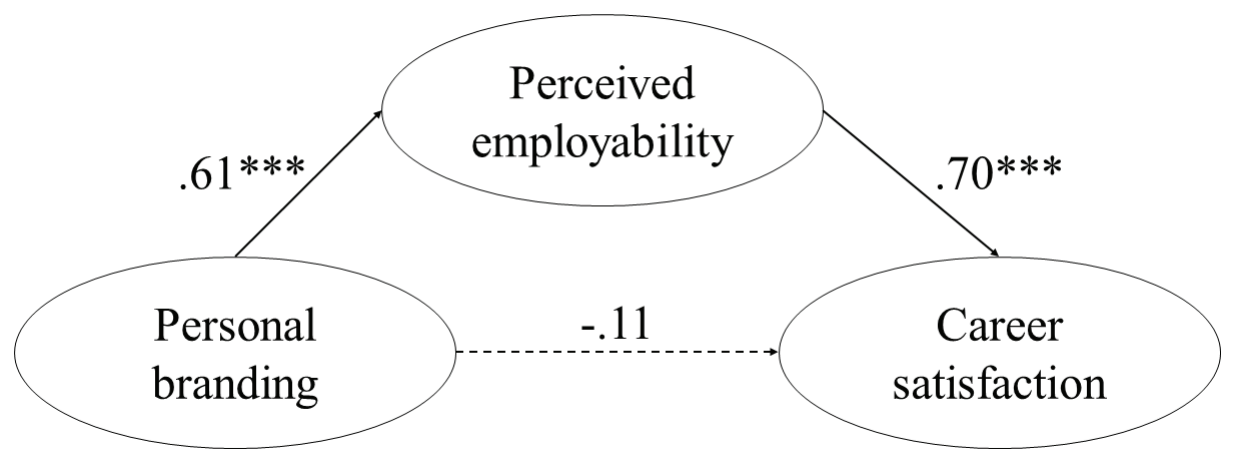
Final mediation model showing the positive effect of personal branding on career satisfaction is mediated by one’s perceived employability (Study 1). Regression results are reported as standardized betas. ^***^p < 0.001. This model explains 43% of the variance [*R*^2^ = 0.43, 95% CI (0.24–0.61)].

We proposed that personal branding is positively related to perceived employability and career satisfaction *via* perceived employability. In line with these hypotheses, the SEM results indicated that personal branding positively and significantly related to perceived employability (*γ* = 0.61, *p* < 0.001), and perceived employability positively and significantly related to career satisfaction (*β* = 0.70, *p* < 0.001); the relationship between personal branding and career satisfaction when accounting for perceived employability, however, was non-significant (*γ* = −0.11, *p* = 0.34). The model indicated a significant indirect effect of personal branding on career satisfaction through perceived employability [*indirect effect* = 0.63, 95% BCa CI (0.36; 1.16), *p* < 0.001], as graphically represented in Figure 1. Thus, Hypotheses 1a–1c were supported.

### Discussion

The findings of Study 1 show that, in line with our hypotheses, personal branding had a positive and significant indirect effect on career satisfaction *via* perceived employability. It means that, by itself, personal branding does not impact satisfaction with one’s career. However, personal branding implies taking proactive career-enhancing steps and clarifying the desired professional future self in the future (Strauss et al., 2012). This is positively related to perceived employability, which, in turn, has been proven to lead to greater career satisfaction.

## Study 2

Having established the positive relationship of personal branding and perceived employability, the aim of the second study was to focus on the antecedents of personal branding. Given that the relationship between perceived employability and career satisfaction is well studied and described in many papers (De Vos et al., 2011; Dacre Pool and Qualter, 2013; Lo Presti et al., 2018), we left only perceived employability as the outcome of personal branding for the sake of model simplicity.

To understand the reasons why individuals may engage in personal branding, we framed its antecedents in the theory of planned behavior (TPB; Ajzen, 1991). The TPB posits that, in order for a behavior to be performed, three determinants of intention must be satisfied: attitude, subjective norm, and perceived behavioral control (Ajzen, 1991). These determinants strengthen or weaken the *behavioral intention*, which, in turn, predicts the enactment of that behavior.

First, the attitude toward engaging in personal branding to achieve greater career success must be positive. Such attitude is encapsulated in the concept of *career achievement aspiration* (Gregor and O’Brien, 2016). While some authors allow a possibility of personal branding for other purposes, such as dating (Labrecque et al., 2011), the literature conclusively suggests that individuals are more likely to engage in personal branding when they perceive a career-related benefit; those who are motivated by advancing own career are more likely to use personal branding as a career tool. Gregor and O’Brien (2016) suggested achievement, leadership, and educational factors of career aspiration, but, given the diversity of career experiences where individuals may apply personal branding, we focused only on career achievement aspiration.

Second, the subjective norm refers to the social pressure on the individuals to progress in their careers. Getting improvement feedback is known to lead to a variety of positive career outcomes, such as job performance, organizational citizenship behaviors, (Whitaker and Levy, 2012), and job satisfaction (Anseel and Lievens, 2007). Hence, getting feedback on how someone should go about positioning herself professionally should increase the intention to engage in personal branding.

Third, perceived behavioral control, such as an individual’s beliefs about the ease or difficulty of performing a particular behavior, is theorized in our research as *career self-efficacy* (Day and Allen, 2004). When an individual feels in charge of his/her own career and feels able to execute the desired career behaviors well, the likelihood of engaging in personal branding increases. Ajzen (1991) posited that “perceived behavioral control, together with behavioral intention, can be used directly to predict behavioral achievement” (p. 184). This makes us conclude that career self-efficacy combined with the intention to engage in personal branding will lead to doing so, and career self-efficacy will also have a direct effect on personal branding.

While thinking about doing something is not the same as the action itself, Ajzen and Fishbein (1980) asserted that the best predictor of engaging in a behavior is the intention to do so. We, therefore, hypothesized that people would engage in personal branding if they have a strong intention to do so.

*Hypothesis 2*: (a) career achievement aspiration, (b) career feedback, and (c) career self-efficacy are positively related to personal branding intention.

*Hypothesis 3*: Career self-efficacy is positively related to personal branding.

*Hypothesis 4*: Personal branding intention is positively related to personal branding.

*Hypothesis 5*: Personal branding intention mediates the relationship between (a) career achievement aspiration, (b) career feedback, and (c) career self-efficacy, and personal branding.

The role of self-efficacy, proactive personality, personal initiative, and feedback seeking in driving proactive behaviors has been extensively discussed (Crant, 2000). We hypothesized that similar concepts, such as those studied in this paper, would have the same mechanisms of action when applied to proactive career behaviors, such as personal branding. And, as established in Study 1, personal branding is strongly related to perceived employability. We, therefore, expected that its antecedents would have a positive indirect effect on perceived employability too.

*Hypothesis 6*: Personal branding intention and then personal branding sequentially mediate the relationship between (a) career achievement aspiration, (b) career feedback, and (c) career self-efficacy and perceived employability.

### Method

#### Participants

Participants were recruited *via* the researchers’ networks, e.g., LinkedIn and WeChat, popular in China, and were encouraged to ask their colleagues to also participate using a standardized invitation about the project and a link to the anonymous survey. A total of 249 responses were collected. Similar to the Study 1 data cleansing strategy, after the visual and boxplot analyses, 35 responses were removed because of acquiescing responding or missing data in core variables, resulting in an analyzable sample of 214 cases, containing no missing data (female = 65.4%; Mean Age = 36.7 (SD = 11.40); China = 88.8%, the Netherlands = 6.5%, Germany = 1.9%; bachelor’s degree = 57.5%, master’s degree = 15.9%, high school = 11.2%, college = 10.3%, Ph.D. = 3.3%, secondary school = 1.9%; employed full-time = 75.7%, employed part-time = 5.1%, not employed, looking for work = 13.6%, not employed, not looking for work = 2.3%; a total of 38% had 5 years of work experience or less).

#### Measures

The survey items were translated into Mandarin Chinese following the back-translation procedure (Sperber et al., 1994). The only exception was the career feedback scale, the original version of which was provided to us already in Chinese by the scale authors. Responses to all the statements in this study were provided on a five-point scale ranging from 1 (*strongly disagree*) to 5 (*strongly agree*).

##### Personal Branding

We used the same 18 items as in Study 1 to assess personal branding. Chronbach’s alpha was 0.90. The alphas for the three factors of the scale (strategic, differentiated, and technologically savvy) were 0.82, 0.76, and 0.88, respectively. As we were interested in overall personal branding behavior rather than its subfactors, we chose to stay at the higher scale level.

*Personal branding intention* was measured with two modified items similar to the ones used in a study of pro-environmental behavior based on the TPB (de Leeuw et al., 2015). The items “I am determined to engage in personal branding behaviors on a regular basis” and “I have the will to engage in personal branding behaviors on a regular basis” were sufficiently highly correlated to demonstrate the stability of this scale (*r* = 0.82, *p* < 0.001). We provided the definition of personal branding, used in this paper, to the respondents before they answered these questions.

##### Perceived Employability

We employed the same five-item scale to assess perceived employability. Cronbach’s alpha was 0.89.

*Career achievement aspiration* was measured with a six-item scale developed by Kim et al. (2016) for their studies of college women in Korea. As they voiced concerns around using reverse-scored items in studies in intercultural context, we chose to follow their advice to use a shorter scale vs. the original eight-item scale (Gregor and O’Brien, 2016) as it demonstrated good reliability and validity in that Korean study. An example item was “I plan to obtain many promotions in my organization or business.” Cronbach’s alpha was 0.87.

*Career feedback* was measured with the four-item career improvement subscale of the career goal feedback scale (Hu et al., 2017). A distinguishing feature of this scale was that the items were negatively worded, and hence were reverse scored for the analysis. An example item was “I do not get helpful advice from others about how I can reach my career goals.” Cronbach’s alpha was 0.91.

*Career self-efficacy* was measured by the seven-item scale developed by Dobrow and Higgins (2005). An example item is “I believe that I can do what I need to do in order to make my career successful.” Cronbach’s alpha was 0.92.

#### Analytical Strategy

The model in Figure 1 was tested in two steps, similar to the strategy of analysis employed in Study 1. There were differences in how we executed Step 2. We performed the SEM analysis on a partial disaggregation model (Bagozzi and Edwards, 1998) by creating parcels of items theoretically related to each other as suggested by Little et al. (2013). A large number of items can cause parameter instability related to the possibility of multiple solutions, cross-loadings, and correlated residuals, especially in a small sample such as ours (Little et al., 2002). Parceling results in more stable model solutions, improves the variable-to-sample ratio, remedies small sample sizes, decreases the likelihood of correlated residuals and dual factor loadings, and reduces Type I errors in the item correlations (Bagozzi and Edwards, 1998; Little et al., 2013). To estimate the indirect effects and mitigate the impact of multivariate non-normality of the data, we used the same bootstrapping procedures as in Study 1.

### Results

#### Measurement Model

In order to test the factor structure of our model, we tested several measurement models with the parcels tapping the six latent variables (career achievement aspiration, career feedback, career self-efficacy, personal branding intention, personal branding, and perceived employability). Since some of the alternative models had comparable fit indices and degrees of freedom, we employed Akaike’s information criterion (AIC) index for the proposed and alternative models (Bozdogan, 1987). The AIC is useful for model comparison as it favors the more parsimonious models, while providing no information on the fit of a particular model. In general, the model with the lowest AIC is considered to have the best fit. As shown in Table 4, the measurement model with six latent factors showed the best fit to the data and was therefore chosen for further analyses. All the items had significant loadings on the intended factors (range *λ* = 0.64–0.94, *p*’s < 0.001).

**Table 4:** Goodness of fit and comparative indices of the proposed and alternative measurement models (Study 2).

Model	*χ*^2^	df	*χ*^2^/df	CFI	TLI	SRMR	RMSEA	AIC
Six-factor	188.07	89	2.11	0.96	0.94	0.05	0.07^*^	282.07
Five-factor	270.20	94	2.87	0.93	0.90	0.07	0.09	354.20
Four-factor	533.30	98	5.44	0.81	0.77	0.10	0.14	609.30
Three-factor	688.00	101	6.81	0.75	0.70	0.11	0.17	758.00
Two-factor	763.43	103	7.41	0.72	0.67	0.11	0.17	829.43
One-factor	882.77	104	8.48	0.67	0.62	0.11	0.19	946.78

χ^2^ values are at p < 0.001; RMSEA values are at p_close_ < 0.001 except ^*^p_close_ = 0.007. All the chi-square differences against the baseline six-factor model are significant at p < 0.001.

#### Descriptive Statistics

Table 5 presents the variables’ means, standard deviations (SD), correlations, and reliability measures of the scales. Personal branding, as expected, was highly and significantly correlated with other career-related constructs: perceived employability (*r* = 0.60, *p* < 0.001), career achievement aspiration (*r* = 0.57, *p* < 0.001), and career self-efficacy (*r* = 0.56, *p* < 0.001).

**Table 5:** Study 2 variables’ means (*M*), standard deviations (SD), and correlations.

	*M*	SD	1	2	3	4	5	6
1. Personal branding	3.47	0.59	**0.90**					
2. Personal branding intention	3.38	0.94	0.62^**^	**0.82**				
3. Perceived employability	3.56	0.70	0.61^**^	0.58^**^	**0.89**			
4. Career achievement aspiration	3.77	0.72	0.57^**^	0.55^**^	0.71^**^	**0.87**		
5. Career feedback	2.84	0.94	−0.25^**^	−0.31^**^	−0.24^**^	−0.09	**0.91**	
6. Career self-efficacy	3.81	0.60	0.56^**^	0.46^**^	0.64^**^	0.63^**^	−0.19^**^	**0.92**

*N = 214. The Cronbach’s alphas are displayed in bold on the diagonal*.

** *p < 0.001*.

#### Hypotheses Testing

**Figure 2:**
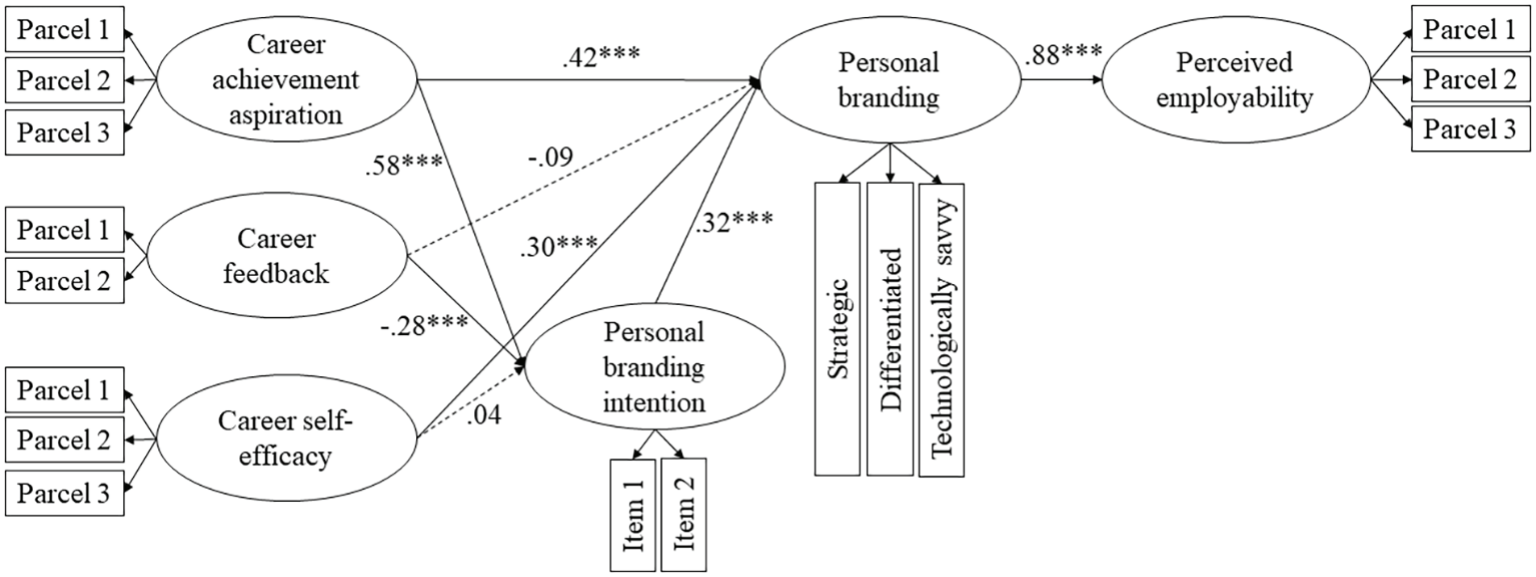
Maximum likelihood estimates for the personal branding model. Solid lines indicate significant paths; dashed lines indicate non-significant paths. Standardized beta weights are reported. *N* = 214. ^***^p < 0.001.

To identify the best model for the analyses, we compared the fit of several theoretically plausible models. Model 1 tested the originally hypothesized relationships as depicted in Figure 2. In Model 2, we tested the full mediation model between career self-efficacy and personal branding. In Model 3, we added direct paths from all the antecedents to personal branding. In Model 4, we removed a direct path in between career feedback and personal branding. In Model 5, we tested full mediation between all the antecedent variables and personal branding. As we see from the results of the models testing shown in Table 6, Model 3 demonstrated both the lowest AIC and better fit indices across the baseline and the alternative models tested, and it was significantly different from the baseline model (Δ*χ*^2^ = 19.24, df = 2, *p* < 0.001). Hence, we proceeded with testing the model represented in Figure 2.

**Table 6:** Goodness of fit and comparative indices of the proposed and alternative models (Study 2).

Model	*χ*^2^	df	*χ*^2^/df	CFI	TLI	SRMR	RMSEA	AIC
Model 1	245.23	95	2.58	0.94	0.92	0.06	0.08	327.23
Model 2	307.40	96	3.20	0.91	0.88	0.11	0.10	387.40
Model 3	225.99	93	2.43	0.94	0.93	0.05	0.08	311.99
Model 4	229.16	94	2.44	0.94	0.93	0.05	0.08	313.16
Model 5	307.40	96	3.20	0.91	0.88	0.11	0.10	387.40

*RMSEA values are at p_close_ < 0.001. All the chi-square differences among the four models are significant at p < 0.001, except for the difference between Models 3 and 4 (Δχ^2^ = 3.17, df = 1, p = 0.07)*.

The hypothesized structural model did explain variance in personal branding intention (*R*^2^ = 48.4%), in personal branding (*R*^2^ = 88.1%), and in perceived employability (*R*^2^ = 76.8%). Career achievement aspiration was positively related to personal branding intention (*γ* = 0.58, *p* < 0.001), career feedback was negatively related (*γ* = −0.28, *p* < 0.001), and the relationship between career self-efficacy and personal branding intention was not significant (*γ* = 0.04, *p* = 0.69). Therefore, Hypothesis 2a was supported, while 2b and 2c were not. Career self-efficacy was positively related to personal branding (*γ* = 0.30, *p* < 0.001), supporting Hypothesis 3. The analyses provided support to Hypothesis 4 that personal branding intention is positively related to personal branding (*β* = 0.32, *p* < 0.001). Testing the mediating effects of personal branding intention, we found that career achievement aspiration had a significant indirect effect on personal branding [*indirect effect* = 0.11, 95% CI (0.11; 0.44)]. The indirect effect of career feedback was significant but negligible [*indirect effect* = −0.04, 95% CI (−0.08; −0.01)], and that of career self-efficacy was not significant [*indirect effect* = 0.01, 95% CI (−0.05; 0.06)]. Thus, Hypotheses 5a and 5b were supported, while Hypothesis 5c was not. Estimating the effects of sequential mediation between the antecedents and perceived employability, we found that career achievement aspiration indirectly positively influenced perceived employability *via* personal branding intention and personal branding [*indirect effect* = 0.16, 95% CI (0.08; 0.27)], while career feedback had a negligible negative effect [*indirect effect* = −0.06, 95% CI (−0.11; −0.02)] and career self-efficacy had a non-significant effect [*indirect effect* = 0.01, 95% CI (−0.07; 0.08)]. These results supported Hypothesis 6a and did not support Hypotheses 6b and 6c.

Additionally, we estimated the indirect effects of career achievement aspiration and career self-efficacy on perceived employability *via* personal branding. The results indicated significant positive relationships: *indirect effect* = 0.36, 95% CI (0.15; 0.63) and *indirect effect* = 0.30, 95% CI (0.04; 0.46), respectively.

### Discussion

The purpose of Study 2 was to examine the antecedents of personal branding. Career achievement aspiration was the strongest predictor of the personal branding intention. Thus, the attitudinal disposition, as explained by the TPB, was the leading indicator for the personal branding behavior. Career achievement aspiration was also strongly related to personal branding, eventually leading to greater perceived employability, confirming the importance of attitudinal disposition for proactive career behavior.

We observed that the societal norm around personal branding has not been settled yet, especially outside the Western contexts (Phua and Caras, 2008; Saleem and Iglesias, 2015), which could explain the negative relationship between career feedback and personal branding intention. Those who receive a lot of career advice (and, therefore, enjoy career help from own network) may have a lower need to engage in personal branding. Our results were consistent with previous studies: a negative relationship was found between feedback on improvement needed and career exploration (Hu et al., 2018), and a positive relationship was found between negative career feedback and career goal disengagement and lowering career goals (Hu et al., 2019). Additionally, we can suppose that people receive and act upon career feedback from more experienced contacts who were likely to become successful in the traditional career models. Therefore, it is plausible to suppose that personal branding is not career advice that people get, and since ignoring the advisors’ recommendations carries relational penalties for the seekers (Blunden et al., 2019), they do not engage in personal branding as an action competing for time and resources to whatever other advice is received.

Lastly, personal branding is still an emerging career competence (Gorbatov et al., 2018) requiring specific competencies, such as technological, metacognitive, creative, and critical skills (Lorgnier and O’Rourke, 2011). Yet, career success still can be achieved *via* traditional mechanisms, especially within organizations (McDonald and Hite, 2005). This could explain the non-significant relationship between career self-efficacy and the personal branding intention (it was measured with two items specifically asking about the intent to perform personal branding activities). However, given significant indirect effect of career self-efficacy on perceived employability through personal branding, we can conclude that people do engage in personal branding but may not call it by that term.

## General Discussion

To better understand predictors and outcomes of personal branding, we conducted two studies, drawing on the contemporary career theory (Arthur, 2008), proactive behavior literature (Crant, 2000; Seibert et al., 2001a), and the TPB (Ajzen, 1991). The studies tested the antecedents and outcomes of personal branding, providing quantitative evidence for its important role for individual career success in the context of contemporary work environment.

### Theoretical Implications

With this paper, we attempted to expand our collective knowledge of proactive career behaviors, such as personal branding, in the context of contemporary work relations. As the notion of career success changes to be seen as a dynamic, context-dependent social construction (Dries et al., 2008), we tried to address the need to examine the relationship between the contemporary view of career success and personal branding that has become “a prominent feature of the labor market, whether in face-to-face settings or in online platforms” (Vallas and Christin, 2018, p. 12). We were inspired by prior research to do so: Roberts (2005) indicated that further research was needed on the “bottom-up tactics” in today’s work environment, Wang and Wanberg (2017) specifically called for more empirical studies of the consequences of engaging in the “gig economy,” while Sullivan and Baruch (2009) urged to extend the career research beyond the Western context.

We also hoped to advance the career theory by examining the ontology of the relationships between personal branding and other career phenomena. In application of the TPB, we focused on the individual drivers leading to personal branding. Earlier research identified other attitudinal antecedents for constructing a positive personal reputation, such as desire for rewards or need to belong (Zinko and Rubin, 2015). Our findings that the attitudinal predisposition, namely career achievement aspiration, was the principal antecedent to personal branding in our study adds to the understanding of why people engage in personal branding. In both studies, personal branding was positively related to perceived employability and career satisfaction, both of which are measures of career success (Boudreau et al., 2001; Arthur et al., 2005; Ng et al., 2005; Greenhaus et al., 2008).

Finally, by providing a generic, reliable, and valid scale to measure personal branding we hope to encourage other scholars in the field to partake in personal branding research. Given the changes in the way people work today that we mentioned in the introduction, more quantitative research is needed to understand how workers and job seekers construct, package, and present their work identities to the target audiences.

### Limitations and Future Research Directions

Like most research, this study had several limitations. First, although the mediation effects found in Study 1 were in line with the extant research (e.g., Dacre Pool and Qualter, 2013), the data in both studies were cross-sectional, thus precluding us from claiming causal inferences and being more susceptible to common method bias. All our three samples relied on the same methodology: self-report surveys. We did our best to mitigate this limitation by conducting the studies in different cultural settings and testing alternative models, which showed a worse fit than the mediation models. Further longitudinal and experimental research is needed to examine the causal nature of the personal branding-career satisfaction relationship, while at the same time accounting for the common method bias. Adding alternative sources of data, such as supervisor assessment or recruiter evaluation, will provide valuable insights on the effectiveness of personal branding.

A second limitation of our study was that the organizational context was out of its scope. Gorbatov et al. (2018) provided a list of work fields ranging from most to least conducive to personal branding, signaling that such activities may develop differently in diverse industry and firm settings. The professional role should also be accounted for, as, for example, freelance workers are more likely to engage in personal branding activities (Gandini, 2016). The context in which certain behaviors take place typically serves as a moderator (see, e.g., Sully De Luque and Sommer, 2000) or a mediator (see, e.g., Liden et al., 2014). Therefore, it is highly advisable that future research explore such moderating and/or mediating effects of the context, in which personal branding occurs.

A third limitation was that we explored only the positive consequences of personal branding for individual career seekers. However, several authors highlighted the “dark side” of personal branding, such as personal branding failures (Labrecque et al., 2011), duress associated with the pervasive pressure to engage in personal branding (Vallas and Cummins, 2015; Vallas and Christin, 2018), pushing the ethical boundaries of the professional field (Cederberg, 2017), commodification of reflexivity (Wee and Brooks, 2010), losing personal identity (Holton and Molyneux, 2017), or, refusing to do so, failing to fit the organization sufficiently to produce a meaningful impact (Shepherd, 2005; Sturdy and Wright, 2008). Future studies should investigate the deleterious impacts of personal branding for individuals, teams, and organizations.

### Practical Implications

Since personal branding, as a contemporary career behavior, in both studies demonstrated strong relationships with career success, workers, job seekers, and employers, labor market intermediaries should invest in understanding what it means to them. For individuals, there is sufficient evidence that personal branding leads to a variety of beneficial outcomes, such as enhanced credibility, visibility, prestige, promotions, or monetary rewards (Gorbatov et al., 2018). Whether organizations benefit from having employees actively engaging in personal branding is still a matter for further research. For students, personal branding could help in the university-to-work transition by contributing to their career identity (Santisi et al., 2018). Finally, the personal branding scale could be a useful diagnostic instrument in a diversity of contexts, such as in training courses aimed to help the participants obtain a deeper insight into career decision-making.

## Data Availability Statement

The datasets generated for this study are available on request to the corresponding author.

## Ethics Statement

Ethical review and approval was not required for the study on human participants in accordance with the local legislation and institutional requirements. Written informed consent from the participants was not required to participate in this study in accordance with the national legislation and the institutional requirements.

## Author Contributions

SG is a Ph.D. candidate, who is the main author of the submitted paper. SK and EL are Ph.D. supervisors who helped SG design the studies. SG did the initial analysis of the literature, was responsible for all the data collection and analysis, and wrote the initial draft. In the consequent process, SK and EL helped to develop the paper toward the final submission.

### Conflict of Interest

The authors declare that the research was conducted in the absence of any commercial or financial relationships that could be construed as a potential conflict of interest.
